# A 12-year analysis of closed medical malpractice claims of the Taiwan civil court: A retrospective study

**DOI:** 10.1097/MD.0000000000010237

**Published:** 2018-03-30

**Authors:** Chi-Yuan Hwang, Chien-Hung Wu, Fu-Cheng Cheng, Yung-Lin Yen, Kuan-Han Wu

**Affiliations:** Department of Emergency Medicine, Kaohsiung Chang Gung Memorial Hospital, Chang Gung University College of Medicine, Niaosong Township, Kaohsiung County, Taiwan.

**Keywords:** civil, claims, medical litigation, medical malpractice

## Abstract

Malpractices lawsuits cause increased physician stress and decreased career satisfaction, which might result in defensive medicine for avoiding litigation. It is, consequently, important to learn experiences from previous malpractice claims. The aim of this study was to examine the epidemiologic factors related to medical malpractice claims, identify specialties at high risk of such claims, and determine clinical which errors tend to lead to medical malpractice lawsuits, by analyzing closed malpractice claims in the civil courts of Taiwan.

The current analysis reviewed the verdicts of the Taiwan judicial system from a retrospective study using the population-based databank, focusing on 946 closed medical claims between 2002 and 2013.

Among these medical malpractice claims, only 14.1% of the verdicts were against clinicians, with a mean indemnity payment of $83,350. The most common single specialty involved was obstetrics (10.7%), while the surgery group accounted for approximately 40% of the cases. In total, 46.3% of the patients named in the claims had either died or been gravely injured. Compared to the $75,632 indemnity for deceased patients, the mean indemnity payment for plaintiffs with grave outcomes was approximately 4.5 times higher. The diagnosis groups at high risk of malpractice litigation were infectious diseases (7.3%), malignancies (7.2%), and limb fractures (4.9%). A relatively low success rate was found in claims concerning undiagnosed congenital anomalies (4.5%) and infectious diseases (5.8%) group. A surgery dispute was the most frequent argument in civil malpractice claims (38.8%), followed by diagnosis error (19.3%).

Clinicians represent 85.9% of the defendants who won their cases, but they spent an average of 4.7 years to reach final adjudication. Increased public education to prevent unrealistic expectations among patients is recommended to decrease frivolous lawsuits. Further investigation to improve the lengthy judicial process is also necessary to relieve the stress of medical malpractice claims on clinicians and practitioners, as well as on the judicial system and rightful claimants.

## 1 Introduction

The incidence of medical malpractice claims is a steadily growing issue in Taiwan because of its impacts at many levels.^[1–3]^ At the practitioner level, lawsuits lead to increasing burnout, decreasing quality of life, and decreasing career satisfaction.^[4]^ Concerns over and experiences of liability suits are associated with changes in clinical practice,^[5,6]^ and these negative influences have expanded to the healthcare system, resulting in possible inadequacies in physician supply in high-risk subspecialties.^[7]^ At the social level, the fear of litigation has also hampered the opportunity to improve patient safety due to physicians’ underreporting of adverse events to reporting systems.^[8]^ Moreover, the high prevalence of defensive medical practice decisions in high-liability specialties leads to the over-ordering of medically unnecessary tests,^[9–11]^ further increasing the monetary burden both directly on self-financing patients and indirectly upon taxpayers.

To implement changes that would reduce malpractice litigation, it is important to learn from previous malpractice claims that offer insight into litigious errors in clinical practice.^[12]^ Understanding the epidemiology of malpractice claims in primary care could help risk management, improve liability claims exposure, reduce liability payments,^[13]^ and potentially also improve practice quality.^[14]^ However, there are data limitations that challenge such understanding. For instance, Western data were mostly derived from the U.S.'s National Practitioner Data Bank or insurer databases,^[15–18]^ because most paid medical malpractice claims in America were settled by insurers. It is also necessary to analyze closed malpractice claims determined by court verdicts because there might be differences in the characteristics of paid claims compared with those settled out of court. Lack of information on the litigation process, such as the odds of a favorable judgment for the clinician and the length of time required for litigation to be resolved, might also heighten stress for clinicians.

The previous literature on closed court verdicts were limited by their relatively small scale or for the specificity of their subspecialty analysis.^[19–21]^ Using Taiwan's population-based national judicial databank, this study aimed to analyze the closed malpractice claims of the civil courts in Taiwan, seeking to explicate the epidemiologic factors of the legal process, identify high-risk diseases, and determine the errors that lead to litigation. With a better understanding of this information, it is believed that clinicians could improve patient safety by avoiding litigious errors, developing educational strategies, and reducing the stress of facing litigation.

## 2 Methods

### 2.1 Study design

Medical malpractice claims are described as lawsuits against physicians, nurses, or healthcare institutions that were filed by a patient or patient's heir for injury or death (respectively) arising from medical care. The present research conducted a retrospective study to review Taiwanese civil court medical malpractice verdicts from 2002 to 2013. The study received approval from the Institutional Review Board at Chang Gung Memorial Hospital.

### 2.2 Study setting and population

The Taiwanese Ministry of Justice maintains a national electronic, de-identified database, “The Judicial Yuan of the Republic of China Law and Regulation Retrieving System,” which includes the judgments in all civil litigation cases that have reached the District Courts, High Courts, and the Supreme Court since 2001.

First, this study researched the civil verdicts of the District Courts from January 2002 to December 2013 inclusive using the keywords “damages” (the compensation basis for malpractice judgments) and “medical,” which include physicians and hospitals in the Chinese language. Second, the study examined the abstracted verdicts to collect the cases involving medical malpractice claims. All the subspecialties except for traditional Chinese medicine and dental practice were included. Third, the malpractice claims were traced in the database to track the appeals process into the High Courts or the Supreme Court. The cases were either concluded by the Supreme Court judgment or the plaintiffs had no appeal after the District Court or High Court adjudication. Trials that were decided before June 30, 2015 were enrolled as the final study cases. Cases dismissed due to incorrect legal procedural process of law, such as violations of statutes of limitation, were excluded from the sample.

### 2.3 Outcome measures

Data documented included the number and specialty of the medical personnel involved, level of involved medical institution, diagnosis and outcome of the alleged injury, final court rendered judgments, results of judgments, indemnity paid (if any), and length of time between incident and litigation closure. The study cases were further categorized according to the specialty involved, comprising:

1. internal medicine (cardiovascular, chest, general medicine, infection, nephrology, gastrointestinal, hematology, oncology rheumatology, metabolic-endocrine, and neurology;
2. surgery (general surgery, neurosurgery, orthopedic, proctology, plastic surgery, cardiovascular surgeon, and anesthesiology);
3. obstetric;
4. gynecology;
5. pediatric;
6. emergency medicine; and
7. others.

The final judgments were made by the Supreme Court, the High Courts, or the District Courts, depending on whether there was any appeal process. If the cases were ever reassigned to a High Court by the Supreme Court, the final judgments were documented as “remanded,” whether the final judgment was made by High Court or Supreme Court. Four levels of hospital classification were used, comprising medical centers, regional hospitals, district hospitals, and clinics, based on the Taiwanese accreditation system.

The outcomes of injuries were categorized into five severity levels, comprising, in ascending order of severity:

1. other injury, including emotional injury, chronic pain, or unsatisfied cosmetic outcome without physical function loss;
2. temporary injury (curable physical injury but needing repeated surgery or a prolonged in-patient stay, such as retaining a surgical needle during a hysterectomy, resulting in repeated surgery);
3. permanent injury (loss of organ, limb/organ dysfunction, or limb amputation);
4. grave injury, such as a brain injury that causes a vegetative state; and
5. death.

In pregnant patients with both maternal and fetal injury, the more severe one was used for classification.

“Primary dispute” is defined as the plaintiff's single most significant argument in the litigation in this study. The type of primary dispute was categorized into 7 major groups, comprising diagnostic error, performance error, surgery related, procedure complications, pregnancy related, drug side effect, and patient security. First, pregnant patients were all categorized into the group “pregnancy related” and divided into 3 subgroups, comprising: undiagnosed congenital anomaly, perinatal complications (e.g., amniotic embolism and dystocia), and other diseases during pregnancy (e.g., appendicitis). Second, the cases involving disputes related to surgery were categorized into the “surgery-related” group and divided into 3 subgroups: direct surgical complications (e.g., ureter injury during hysterectomy), disease occurring in post-operation care (e.g., wound infection or post-operative nosocomial infection), and unsatisfied surgical result (e.g., unsatisfied cosmetic effect or persistent pain after spine operation). Third, disputes over nonsurgical invasive medical procedures—such as central venous catheter access, alimentary scope exams, or angiographies—were all categorized into the “procedure complications” group. In the remaining cases, diagnosis errors were those in which the initial diagnosis differed from the final disease that caused the alleged injury. Performance errors were those in which the diagnosis was correct, but the condition was treated in an inappropriate manner, including failure to perform an indicated treatment, inappropriate medication dosage, or failure to order consultation or refer. In the group “patient security,” the healthcare provider's failure to offer adequate safety support or monitoring resulted in a mentally deficient patient committing suicide, falling, or removing tubes by themselves. The categories of primary dispute were determined by the 2 co-authors of this study independently, after each reviewed all of the verdicts. The final decision in inconsistent cases was made through a consensus meeting with a third reviewer.

### 2.4 Data analysis

Descriptive statistics were used to evaluate the data. Data were presented as mean ± standard deviation (SD) or median ± inter-quartile range and percentages (%), and analyzed by student *t*-test. The indemnity amount was presented in US dollars, with an exchange rate (based on the average over the past few years) of 30:1 to New Taiwan dollars (NTD).

## 3 Results

**Figure 1:**
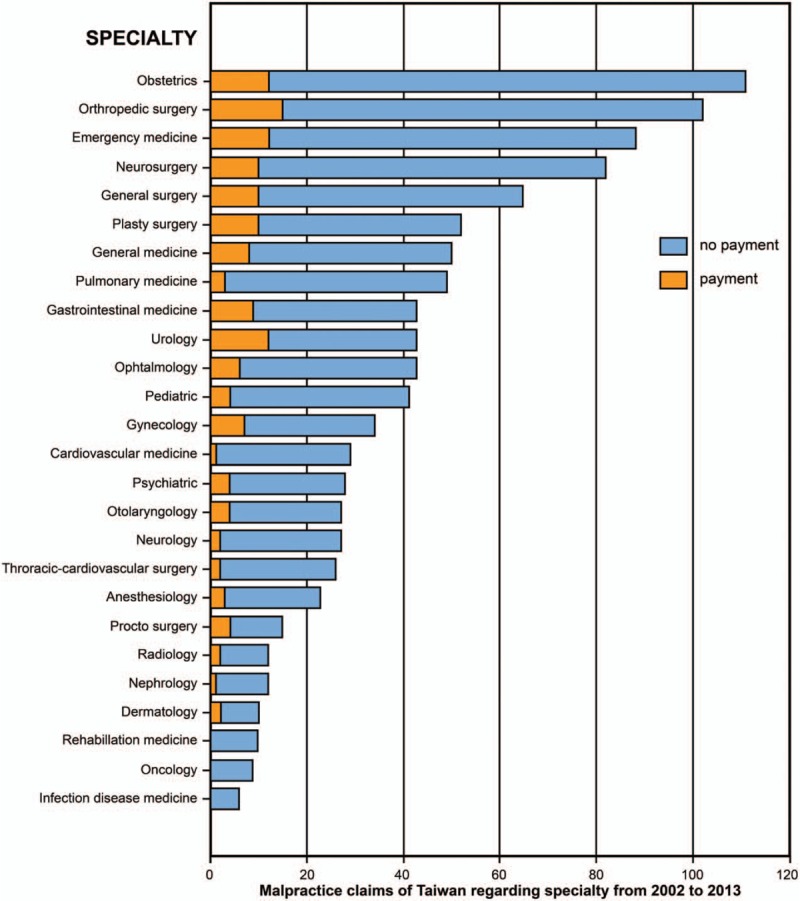
The number of malpractice claims and cases with indemnity paid regarding the medical specialties.

All 9359 verdicts extracted from the District Court's database between January 1, 2002 and December 31, 2013 were retrospectively reviewed. In total, 967 closed medical malpractice claims were identified their appeals processes traced. Twenty-one cases were excluded, as they were dismissed due to incorrect legal process. The remaining 946 closed verdicts comprised the study group. A hospital was the only defendant in 95 cases, but there were 1037 physicians, 112 resident physicians, and 113 nurses sued in the remaining 851 cases. The numbers of physicians who were filed against and ordered to pay an indemnity by each subspecialty are shown in Figure 1. The top 3 specialties involved in malpractice claims were obstetrics (10.7%), orthopedics (9.8%), and emergency medicine (8.5%).

The basic demographic data of malpractice claims are shown in Table 1. In total, 133 cases (14.1%) resulted in an indemnity payment, with a mean payment of 83,350 ± $129,629 (median $34,140). Categorized by specialty group, the majority (39.4%) of the study cases were categorized as surgeon, which accounted for 46.3% of indemnity paid. The percentage of paid claims, defined as the number of cases with indemnity paid divided by the total number of claims in a specialty group, was highest in gynecology (18.4%) and lowest in obstetrics (8.4%). In total, 963 hospitals were involved; 2 hospitals were sued in 17 cases. The majority of cases came from larger hospitals, including medical centers (38.6%) and regional hospitals (34.2%), but the percentage of paid claims was highest in clinics (17.1%). More than half of the cases were concluded in the District Courts (57.3%), and appeals were made to the High Court (24.9%) and the Supreme Court (14.8%). The mean length of time between the incident and litigation closure was 56.4 ± 25.9 months. The cases in which indemnity was paid took longer to conclude than cases in which indemnity payment was not ordered, but no statistical significance was found (p = 0.346). The time from incident to judgment was longest in the District Courts (47.6 ± 20.0 months). When cases were appealed, it took the High Courts and Supreme Court additional time to deliver judgments (16.2 ± 10.6 and 6.5 ± 5.1 months, respectively).

**Table 1:**
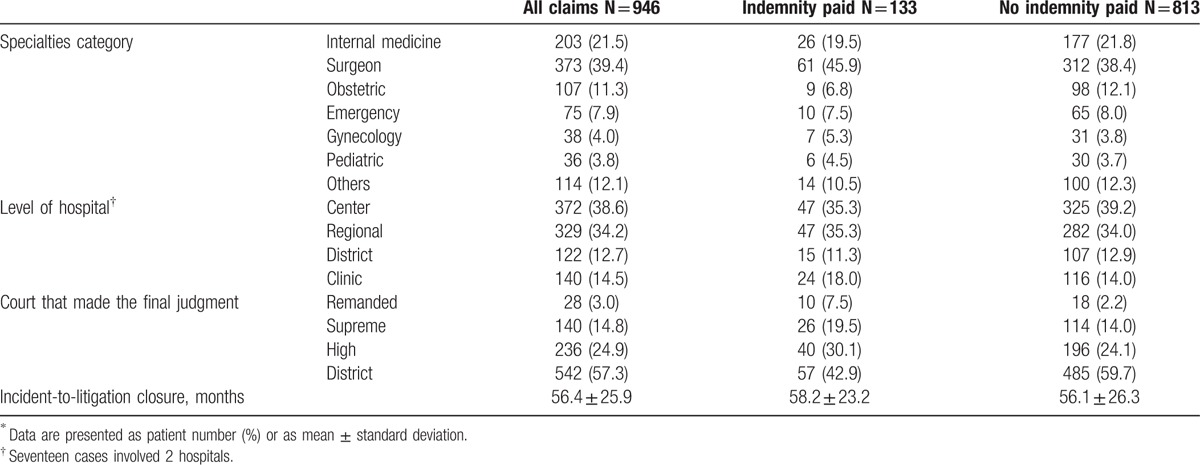
Demographic data and characteristics of malpractice claims, subdivided according to payment of indemnity^∗^.

Indemnity paid divided by patient outcome is shown in Table 2. The majority resulted from tragic outcomes, with 46.3% of plaintiffs having died (36.9%) or experienced grave injury (9.4%). The percentages of paid claims in each subgroup were similar, but the amount of indemnity paid differed. Patients with grave outcomes had the largest mean indemnity amount ($346,328), which was approximately 4.5 times higher than in cases in which the plaintiff died. Patients without permanent physical injury, including temporary injury (16.4%) and other injury (11.0%), accounted for approximately a quarter of the cases, and the indemnity paid was about a quarter of the indemnity paid in cases of deceased patients.

**Table 2:**
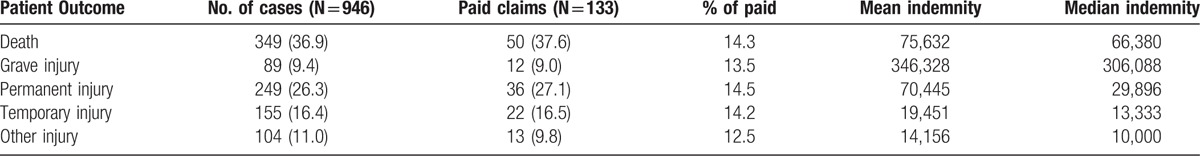
Indemnity paid divided by patient outcome involved in medical claims.

The 10 most common diagnosis groups are presented in Table 3; these account for 41.4% of all cases. Overall, undiagnosed congenital anomalies (4.5%) and infectious diseases (5.8%) had the lowest percentages of paid indemnifications. Spine surgery (4.3%) for spondylosis, herniated intervertebral disc, or spondylolisthesis was the most encountered operation type in malpractice claims. Cosmetic surgery (3.9%), ophthalmologic surgery (3.2%), and gynecologic surgery (2.7%) were also high risk for litigation. Although the highest percentage of paid indemnifications occurred in the cosmetic operation-related group (24.3%), the mean indemnity paid ($11,248) was the lowest.

**Table 3:**
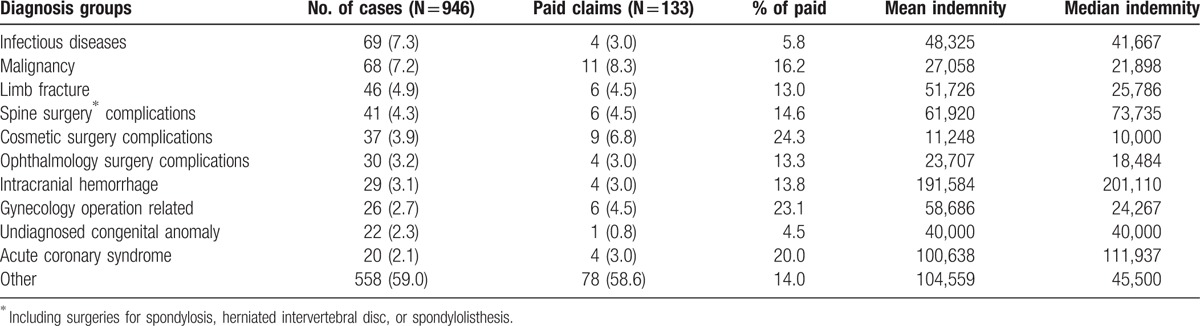
Indemnity paid in top 10 diagnosis groups involved in medical claims.

The categorization of primary disputes in malpractice claims is shown in Table 4. Surgery-related disputes (38.8%) were the most frequent in civil malpractice claims. Perinatal complications (6.6%) and undiagnosed congenital anomalies (2.3%) resulted in 78.5% of malpractice cases for the pregnancy subgroups. Although the number was relative low, the percentage of paid claims was highest (26.7%) in the subgroup of patient security. The lowest percentage of paid indemnity was found in the group of undiagnosed congenital anomalies (4.5%), postoperation care (7.2%), and performance errors (7.3%).

**Table 4:**
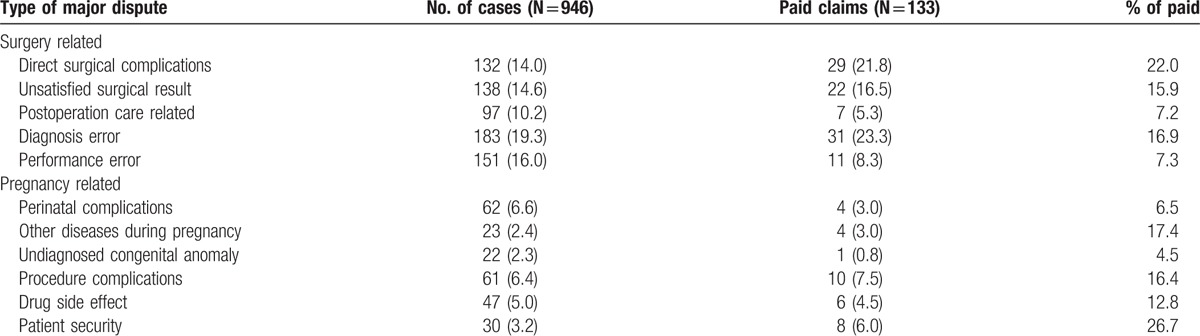
Categorized primary disputes and percentage of paid indemnity in medical malpractice claims.

With further analysis of the relation between the primary dispute and the diagnosis groups, performance error was the primary dispute in in 69.6% of the cases in the infectious group. Diagnosis error mostly occurred in the malignancy (80.9%), intracranial hemorrhage (62.1%), and acute coronary syndrome (70%) groups. Unsatisfied surgical results accounted for approximately half (48.7%) of the causes of litigation in limb fractures, spine surgeries, cosmetics operations, and ophthalmologic surgery.

## 4 Discussion

This study provided analysis of 12 years of population-based information on closed claims, providing insight into non-Western civil court experiences and generating several findings that need to be addressed.

First, the single most risky specialty in our study was obstetrics (10.7%), and surgery accounted for 39.4% of the study cases, with orthopedic, neurosurgery, and general surgery the top 3 specialties. These results are compatible with the prior literature's recognition that obstetrics and surgery are high-risk specialties.^[17,21–24]^ The most commonly litigated surgery types performed by a surgeon in the present study were limb fractures, spine surgery, and cosmetic surgery. Although the average indemnity paid was the lowest ($11,248), the percentage of paid claims was highest (24.3%) in the cosmetic surgery group, within which an unsatisfied surgical result accounted for 83.8% of claims. This analysis suggests that the origin of dispute in these specialties lies in the unrealistic expectations of plaintiffs. Patients always expect perfect medical results, such as safe labor in childbirth, or flawless eyelid surgery. Even when the patient is well-informed of the inherent risks, plaintiffs questioned the alleged “error” when complications or simple imperfections happened. The existing literature focusing on plastic surgery litigation demonstrated that the key elements heavily influencing judicial decisions were the quality of medical records and informed consent, which should include informed consent information, preoperative and postoperative photographs, and information allowing an outside expert to adequately review the chart.^[25]^ This study proposes that these principles be applicable for the aforementioned high-risk specialties to ensure better legal defense in litigation.

Second, the percentage of indemnity paid in different medical injuries was similar, which implies that the court does not judge in favor of the plaintiff just because a tragic outcome occurred. However, the amount of compensation was mostly based on patient outcome, but unrelated to specialty or type of medical error. Using the indemnity for a deceased plaintiff as a baseline, the patients with grave outcomes were awarded, on average, approximately 4.5 times the monetary compensation of other plaintiffs, and the patients without permanent physical injury received around a quarter of the indemnity paid in claims brought on behalf of deceased plaintiffs. The trend that grave injuries had the 2 to 2.5 times the amount of indemnity paid to deceased patients was also found in America and China,^[24,26,27]^ but this disparity might create intolerable moral and economical conflicts: some physicians might, consequently, lack the incentive for aggressive treatment and prefer the outcome of patient death (rather than grave injury) in cases with malpractice risks. Accordingly, the present analysis suggests the need for further discussion on various compensation payments for different injuries to both ensure a fair compensation system and prevent moral conflicts.

Third, compared with the average percentage of paid indemnities (14.1%), it was noteworthy that a relatively low percentage of payment was found in certain diagnosis groups, including undiagnosed congenital anomalies (4.5%) and infectious diseases (5.8%). In previous Taiwanese closed civil claim studies, treatment in 71.2% to 88.5% of the cases was deemed appropriate by the medical appraisal within the litigation process,^[19,20]^ and a similar percentage was expected in the current study. The reason for the plaintiffs’ willingness to spend years in litigation contesting the appraisal result might be attributed to their misunderstanding of medical limitations. Plaintiffs might subjectively believe that certain diseases could be avoided if the clinicians expend more effort. For example, parents might blame obstetrics clinicians for their carelessness in prenatal echo screening when a congenital limb deficiency is later found after birth. Although the limitations of echo procedures are well known, these claims still accounted for 32.7% of fetal related litigation in previous studies.^[20]^ Obstetrics was found to be the riskiest specialty category in this study, but the percentage of paid indemnity was also found to be the lowest. It is also compatible with previous studies that there were no verifiable medical errors in 37% of the study cases,^[22]^ as sometimes plaintiffs file a lawsuit due to malice resulting from their misperceptions. Litigation constitutes an unnecessary economical and judicial waste for both the defendant and the plaintiff where there has been no actual medical error. Increased patient acknowledgement of medical limitations through public education might help to prevent frivolous lawsuits based on misunderstanding and resentment.

Finally, the lengthy judicial process was stressful for clinicians because, in 85.9% of cases, they are required to spend an average of 4.7 years to prove their innocence. Fear of litigation originates from not only the potential monetary loss, but also the time loss, stress, and negative career impacts provoked by malpractice claims. Prior literature has revealed that legislation changing the malpractice standard for emergency physicians had little effect on the intensity of practice measured by imaging rates, average charges, or hospital admission rates.^[28]^ This study suggests that the effect was only partial because those physicians defensively practice not to “win the malpractice claim” but to “avoid the malpractice claim.” Malpractice claims that originate from the plaintiff's perception of unfair medical procedures will never disappear. Besides offering legal protection by tort reform to ensure fair judgments, greater efforts to relieve litigation stress by improving the inefficient judicial process, which is believed to have more impact on clinical behavior, should also be investigated.

### 4.1 Limitations of the study

Several limitations should be noted in this study. First, the study considered closed court verdicts, but cases withdrawn due to court settlements were not available in the database. This might have caused underestimation of the actual number and occurrence rate of malpractice claims. Second, the data were analyzed based on the verdict, so the detailed information reviewed may not be as complete as a medical record, and hence might affect the precision of the dispute analysis. Third, detailed information on the plaintiffs and defendants was not documented in the verdicts, so further analysis of the risk factors of lawsuits was not possible. Finally, analysis of closed medical claims represents only the first step of advancing patient safety, and further intervention studies based on information derived from court verdicts experiences is warranted to address ways to decrease preventable medical errors.

## 5 Conclusions

By analyzing 946 closed civil court verdicts on medical malpractice suits in the national database, this study found that 86% of the verdicts favored the clinicians, but these clinicians had to spend 56.4 ± 25.9 months awaiting the final adjudication. The obstetrics and surgery groups accounted for more than half of the cases. The diagnosis groups at high risk of litigation were infectious diseases, malignancies, and limb fractures. The most frequent subjects of dispute in civil malpractice claims were surgery related followed by diagnosis error. This study proposes further reform to improve the lengthy judicial process to relieve the stress for clinicians involved in malpractice claims.

## Author contributions

**Conceptualization:** K.-H. Wu, Y.-L. Yen.

**Data curation:** K.-H. Wu, C.-H. Wu, F.-C. Cheng.

**Formal analysis:** Y.-L. Yen.

**Investigation:** C.-Y. Hwang.

**Methodology:** C.-H. Wu.

**Resources:** C.-H. Wu.

**Supervision:** K.-H. Wu.

**Writing – original draft:** C.-Y. Hwang.

**Writing – review & editing:** K.-H. Wu.
